# Omega 3 Fatty Acids Supplementation and Oxidative Stress in HIV-Seropositive Patients. A Clinical Trial

**DOI:** 10.1371/journal.pone.0151637

**Published:** 2016-03-25

**Authors:** Norma Amador-Licona, Teresa A. Díaz-Murillo, Genaro Gabriel-Ortiz, Fermín P. Pacheco-Moises, Texar A. Pereyra-Nobara, Juan M. Guízar-Mendoza, Gloria Barbosa-Sabanero, Gustavo Orozco-Aviña, Sandra C. Moreno-Martínez, Rafael Luna-Montalbán, Eduardo Vázquez-Valls

**Affiliations:** 1 Department of Education and Research, UMAE HE No.1, Bajio, Instituto Mexicano del Seguro Social, Mexico City, Mexico; 2 Laboratory of oxidative stress & Pathology, Centro de Investigacion Biomedica de Occidente, Instituto Mexicano del Seguro Social, Guadalajara, Mexico; 3 Department of Chemistry, University of Guadalajara, Guadalajara, Mexico; 4 University DeLa Salle Bajio, Leon, Mexico; 5 Department of Research, University of Guanajuato, Leon, Mexico; 6 Institute of Research on Inflammation, Guadalajara, Jalisco, Mexico; Temple University, UNITED STATES

## Abstract

HIV-seropositive patients show high incidence of coronary heart disease and oxidative stress has been described as relevant key in atherosclerosis development. The aim of this study was to assess the effect of omega 3 fatty acids on different markers of oxidative stress in HIV-seropositive patients. We performed a randomized parallel controlled clinical trial in The Instituto Mexicano del Seguro Social, a public health hospital. 70 HIV-seropositive patients aged 20 to 55 on clinical score A1, A2, B1 or B2 receiving highly active antiretroviral therapy (HAART) were studied. They were randomly assigned to receive omega 3 fatty acids 2.4 g (Zonelabs, Marblehead MA) or placebo for 6 months. At baseline and at the end of the study, anthropometric measurements, lipid profile, glucose and stress oxidative levels [nitric oxide catabolites, lipoperoxides (malondialdehyde plus 4-hydroxialkenals), and glutathione] were evaluated. Principal HAART therapy was EFV/TDF/FTC (55%) and AZT/3TC/EFV (15%) without difference between groups. Treatment with omega 3 fatty acids as compared with placebo decreased triglycerides (-0.32 vs. 0.54 mmol/L; p = 0.04), but oxidative stress markers were not different between groups.

**Trial Registration:**

ClinicalTrials.gov NCT02041520

## Introduction

Increased incidence of cardiovascular disease (CVD) occurs in HIV-infected patients compared with general population [1]. The mechanism of coronary heart disease (CHD) among HIV-infected patients reflects a complex interplay of factors, including traditional risk factors, antiretroviral drug effects, and HIV-related parameters, such as inflammatory and immunologic changes [2,3]. There is increasing evidence that oxidative imbalance lead to increased stress on cellular structures and causes changes in molecular pathways that underpins the pathogenesis of several important human diseases, including heart disease, neurological disease, cancer, and ageing [4,5]. Antioxidant imbalance that is assessed through plasma malondialdehyde concentration and plasma total antioxidant ability is a condition, which can contribute to increased destruction of CD4+ T cells and even disease progression if the balance is in favor of pro-oxidant (free radicals) generation in HIV-infected patients [6,7].

In general population, in patients with diabetes and with rheumatoid arthritis, previous studies have shown that omega-3 fatty acids may reduce inflammation, oxidative stress, and fat mass [8–10], but the results are inconclusive, due, in part, to the type of omega-3 fatty acids used. In hypertriglyceridemic HIV-seropositive patients, a weak anticytoquine effect was observed and triglyceride levels decreased in conjunction with fish oil supplementation [11]. However, there is no information about the effect of omega- 3 fatty acids on oxidative stress in these patients. Thus, the main objective of our study was to evaluate the change on lipoperoxides, nitric oxide catabolites and glutathione levels in HIV-seropostive patients after treatment with omega 3 acids for 6 months. We chose omega-3 fatty acids instead of other fatty acids because highly purified EPA and DHA became available and evidence documenting individual effects of EPA and DHA has been accumulated. For example the very rapid incorporation of omega-3 FAs into cell membranes thus affecting function of cells and tissues with subsequent impact on production of various vasoactive eicosanoids and other mediators [12].

## Material and Methods

### Study design

We performed a randomized, double blind, parallel, controlled clinical trial. The protocol was approved from the scientific and ethic institutional committees on October 25, 2011, and no conflict of interest is specified. This study was registered on Clinical Trials NCT02041520. However; delay in registering this study after enrolment of participants started was secondary to administrative procedures for use of financial resources and documentation at the institution for clinical trials registration. We confirm that all ongoing and related trials in the future will be register before enrolment of participants.

### Sample size

It was calculated according to different oxidative stress markers. For example, for malondialdehyde we consider a previous study in patients on hemodialysis [13] where after 2 months of omega 3 fatty acids treatment, a difference of 0.9 nmol/l was found between groups, with a SD = 0.9 and 0.7 nmol/l in the treatment group and in the control group, respectively. With this information we obtained a sample size of 17 patients per group for a 80% statistical power with α = 0.05. Similar sample size was calculated for glutathione considering a difference = 500 μg/l between groups and SD = 700 μg/l [14]. With this information we obtained a sample size of 31 patients per group, and finally for nitric oxide catabolites, we calculated a sample size of 30 patients per group considering a difference in 25% in its levels between groups and SD = 10μmol/L with similar statistical power and α value [15]. Assuming a 15% patient lost, a sample size of 35 per group was considered. Participants were obtained by consecutive cases who met the inclusion criteria, up to the given sample size.

### Subject Selection

We studied HIV-seropositive patients from 20 to 55 years old, on clinical score A1, A2, B1 or B2 receiving highly active antiretroviral therapy for at least 3 months but protease inhibitors, with CD4> 200 cel/mL and with at least one of the following alterations in lipids: Triglycerides between 200 to 500 mg/dL, LDL cholesterol between 130–160 mg/dL without use of hypolipidemic agents. We excluded patients with diagnoses of co-infections (B or C hepatitis, tuberculosis, etc.), diabetes mellitus, hypertension, use of anticoagulants, dyslipidemia identified before receiving HAART therapy, and use of protease inhibitors.

All those patients who evolve to more advance clinical scores during the following in the study were eliminated from the study.

#### Patients

In the department of infectious diseases of the UMAE No. 1, Instituto Mexicano del Seguro Social, patients who met the inclusion criteria were informed about the study's purpose, procedures, risks and benefits. If accepted for participation, after clarification of all doubts, they signed the informed consent. The study protocol was approved by the Ethical and Scientific National Commission Committee No. 785 of the Instituto Mexicano del Seguro Social with the following reference number: R-2011-785-058. All interventions were in accordance with The Code of Ethics of the World Medical Association (Declaration of Helsinki) for experiments involving humans.

We reviewed all clinical records to determine the characteristics of the disease, the time from the detection of the disease and the presence of complications or co-morbidities. In any case we selected patients using triple therapy but not protease inhibitors. When a patient during follow up showed viral load> 1000 copies, sequence genotype was requested and optimized treatment was assigned if required. Participants who meet the inclusion criteria were randomized 1:1 to either omega-3 fatty acid ethyl esters 2.4 g/day (Zonelabs, Marblehead MA) or placebo (olive oil gelcaps, Perfect Source, Fullerton CA, product code number PER 1016, lot number 8A0019/1600-1) 4 g/day for 6 months of treatment. The randomization list was centralized by the Institute of Research on Inflammation, which assigned the next number available on the list to each newly enrolled patient and informed the local centre’s investigator of the treatment group allocation. Gelcaps in both cases were similar in presentation and 2 were indicated in the morning and 2 at night during or after meals. Adherence to treatment was assessed by counting of tablets according to the following formula:

Adherence to treatment(%)=number of capsules actually taken from the last count/number of tablets should be taken at the same stage x100%.

On the visit as basal stage, general determinations were made about nutritional status according to food intake assessment (using the Food Processor software). Anthropometric measurements were performed (weight, height and BMI). For weight, a calibrated electronic scale portable analog Tanita was used. It has a capacity of 120 kg and precision levels of ± 100 g. Patients were weighed in underwear. All measurements were performed after calibration of instruments, and took place in both groups under similar conditions at the beginning and at the end of the study.

At baseline, 3 and 6 months follow-up, we obtained a blood sample and determined the lipid profile (total cholesterol, triglycerides, LDL, HDL, VLDL). Oxidative stress parameters were determined at baseline and final stage. For the latter purpose lipid peroxidation products, total glutathione levels and nitric oxide catabolites were measured.

When lipid profile revealed the persistence of basal levels or increase in both triglycerides and LDL-cholesterol, statins or fibrates were added for hypercholesterolemia or hypertriglyceridaemia respectively according to the "Executive summary of the third report of the National Cholesterol Education Program (NCEP) Expert Panel on Detection, Evaluation, and Treatment of High Blood Cholesterol in Adults (Adult Treatment Panel III)" [16], or decision of their own physician.

#### Determination of lipid peroxidation products

Serums were tested for lipid peroxidation using a Kit from Oxford Biomedical Res Inc. (product No FR 12). Each measurement was repeated four times. The Kit contains a chromogenic reagent (Nmethyl- 2-phenylindole) which reacts with the lipid peroxidation products malonaldehyde (MDA) and 4- Hydroxyalkenals (4-OHA) at 45°C yielding a stable chromophore with maximal absorbance at a wavelength of 586 nm. Results are expressed as nmol MDA+4-OHA (mg protein)−1.

#### Determination of nitric oxide catabolites (nitrates and nitrites)

Nitric oxide was indirectly quantified because during their metabolism nitrates and nitrites are formed. Nitric oxide release was determined spectrophotometrically by measuring the accumulation of its stable degradation products, nitrite and nitrate. Quantification of these metabolites in serum was done using a commercial package (Calbiochem Nitric Oxide Assay Kit, colorimetric 482650). Briefly, Nitrate to nitrite conversion was done using nitrate reductase. Total nitrite is then determined spectrophotometrically by using the Griess reaction. Results are expressed as μM/mL.

#### Determination of glutathione levels

Total glutathione was assessed by enzymatic recycling procedure in which GSH is oxidized by 5, 5´-ditiobis-2-nitrobenzoic (DTNB) acid and reduced by NADPH in the presence of glutathione reductase. The formation of 2-nitro-5-thiobenzoic (TNB) acid was monitored at 412 nm [17]. Total glutathione content of the sample was determined by comparing the observed value with a standard curve generated from known concentrations of GSH. GSSG sample was determined by the above recycling method. 4-vinylpyridine was used in all samples to eliminate reduced glutathione, leaving only the oxidized form of glutathione as a single test substrate. GSH was calculated by subtracting the total glutathione GSSG.

#### Other determinations

Systemic blood pressure was also measured with the patient in sitting position after resting for 15 minutes. The cuff on the non-dominant arm, which was blown with a mercury sphygmomanometer, was placed. At least two determinations with a minimum difference between them 5 minutes were conducted and the average was considered.

The nutritional assessment was performed using 24-hour recall and food diary 3 days after standardization by researchers. The data were analyzed using the software Food processor SQL at baseline and 6-month stage where daily intake of omega-3 and 6 was also evaluated.

### Statistical analysis

Results are expressed as mean ± SD or as median (95% CI) according to variables’ distribution. The primary objective was to assess change of omega-3 fatty acids compared with placebo, based on difference in oxidative stress markers in the intent-to treat population (patients who were lost to follow-up were censored at the time of their last follow-up assessment). As a secondary objective the same endpoints were assessed in the per-protocol population. The Mann–Whitney *U* test or unpaired Student’s *t*-test were performed to evaluate differences between baseline and final variables in group assigned to omega-3 fatty acids or placebo for variables displaying no normal or normal distribution, respectively. Chi-square test was used for categorical variables. A *p* value < 0.05 was considered significant. All data were analyzed using the Statistics software version 6.0 (Statsoft Inc., Tulsa, OK, USA).

## Results

**Fig 1:**
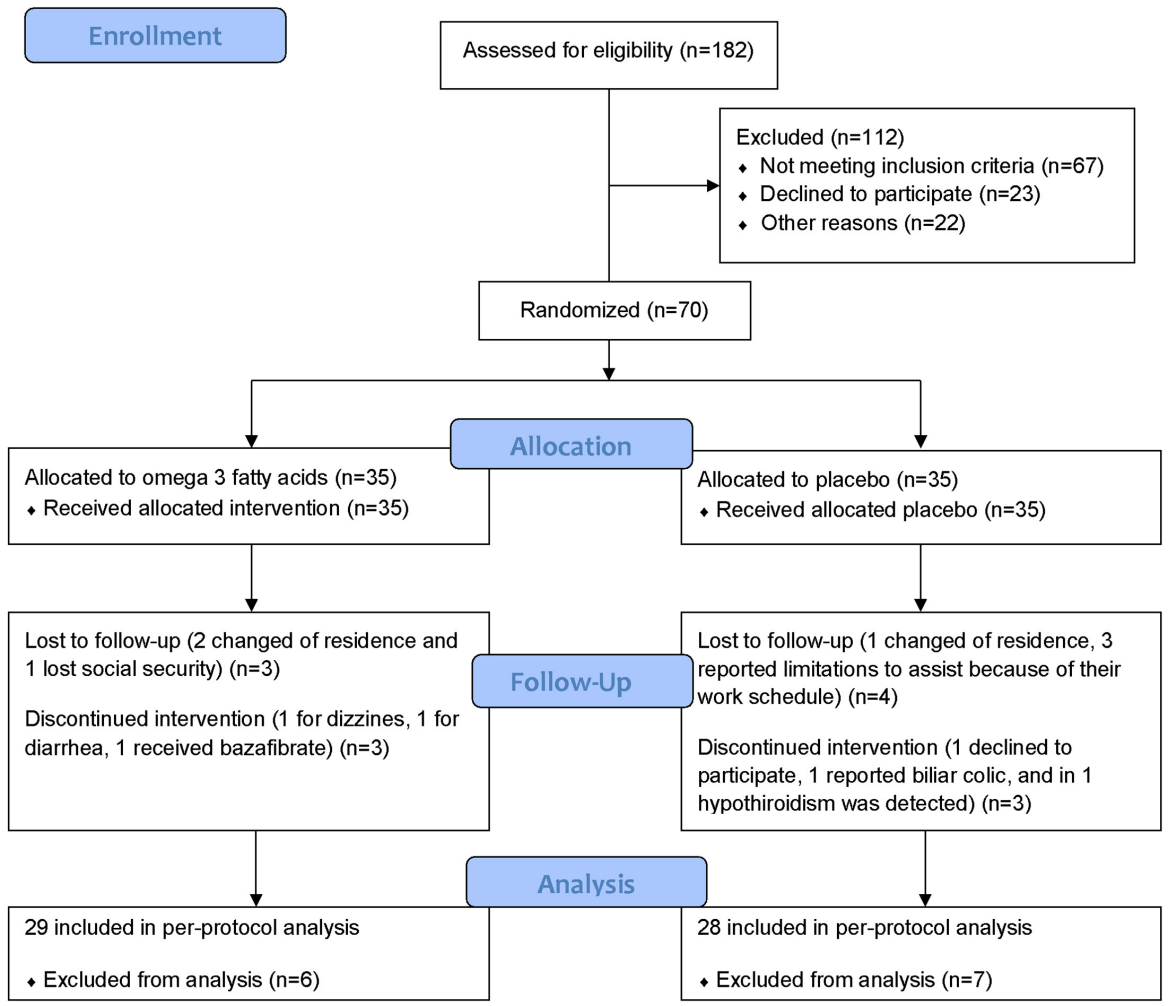
Enrollment, randomization, and follow-up of study patients.

The first patient was recruited in March 2012, with the screening ultimately including 182 potentially eligible HIV-seropositive patients. The enrollment of 70 participants was completed in April 2014. They were followed until they completed the end of the study, withdrew from the study or were lost to follow-up (Fig 1).

Principal HAART therapy was a NNRTI-Based regimen: Efavirez (EFV)/Tenofovir (TDF)/Emtricitabine (FTC) (55%) and Zidovudine (AZT)/Lamivudine (3TC)/EFV (15%) without difference between groups.

The mean rate of adherence to the study drug was 88.2% and 80.4% in the omega group and the placebo group, respectively. Patients who discontinued the study drug prematurely were asked to return for further clinic visits and assessments until the scheduled final visit.

No difference was found in clinical and biochemical characteristics between groups at baseline, including energy, macronutrients, omega-3 and omega-6 acids intake (Tables 1 and 2).

**Table 1:** Baseline clinical and biochemical characteristics between groups. Data are showed as mean ± SD or mean (95% CI) according to normal or not-normal variable’s distribution.

Variable	Omega-3 fatty acids n = 35	Placebo n = 35	p
Gender (M/F)	28/7	23/12	0.18
Age (years)	39.9 ± 9.5	39.9 ± 8.0	0.97
BMI (kg/m^2^)	25.4 ± 4.3	26.5 ± 4.7	0.32
Time from diagnosis (months)	67.2 (51.3–83.0)	81.2 (63.1–99.2)	0.19
Time of treatment (months)	54.3 (40.5–68.1)	64.4 (48.2–80.6)	0.34
SBP (mmHg)	111.7 ± 11.3	113.4 ± 13.2	0.56
DBP (mmHg)	76.2 ± 8.1	73.0 ± 10.6	0.18
Glucose (mmol/L)	5.01 ± 0.39	5.08 ± 0.62	0.60
Total-Cholesterol (mmol/L)	4.9 (4.6–5.7)	5.0 (4.7–5.3)	0.66
Triglycerides (mmol/L)	2.3 (1.8–2.9)	2.0 (1.6–2.4)	0.31
HDL-Cholesterol (mmol/L)	1.2 (1.0–1.3)	1.1 (1.0–1.2)	0.89
LDL- Cholesterol (mmol/L)	2.7 (2.4–3.0)	2.8 (2.6–3.0)	0.68
VLDL- Cholesterol (mmol/L)	1.0 (0.80–1.1)	1.0 (0.80–1.2)	0.77
ALT (UI/L)	40.7 (34.8–46.6)	41.4 (33.5–49.3)	0.89
AST (UI/L)	31.2 (28.5–33.8)	33.3 (25.8–40.8)	0.59
Viral load (copies/ml)	50.0 (nd—977)	50.0 (nd—629)	0.85
CD4 (cel/ml)	526 (438–613)	664 (542–785)	0.10
CD8 (cel/ml)	1259 (972–1546)	1247 (1011–1482)	0.95
CD4/CD8	0.67 (0.30–1.0)	0.65 (0.51–0.80)	0.94
Lipoperoxides (nM/mg protein)	2.5 (2.0–3.1)	2.5 (2.0–3.1)	0.95
Total GSH (μM)	32.7 [15.3–50.1]	22.6 [10.3–34.8]	0.33
GSSG (μM)	10.7 [0.32–21.2]	2.2 [0.11–4.3]	0.28
GSH (μM)	21.9 [9.0–34.2]	20.3 [7.8–32.8]	0.85
NO (μmoles/ml)	36.8 ± 14.2	39.0 ± 13.2	0.51

**Table 2:** Energy intake, macronutrients, n-3 and n-6 fatty acids between groups. Data are showed as mean (95% CI).

Variable	Omega-3 fatty acids n = 35	Placebo n = 35	p
Energy (kcal/día)	2599 (2158–2995)	2612 (2166–3058)	0.96
Proteins (g)	107.2 (88.3–126.1)	110.8 (89.2–132.4)	0.80
Lipids (g)	102.8 (81.9–123.7)	93.6 (76.8–110.4)	0.48
Carbohydrates (g)	313.9 (274.3–353.5)	340.6 (263.1–418.1)	0.53
Omega 3 (g)	1.1 (0.95–1.3)	1.3 (0.82–1.9)	0.43
Omega 6 (g)	20.9 (12.5–28.4)	19.6 (14.5–24.8)	0.78

In the per protocol analysis, treatment with omega 3 fatty acids as compared with placebo decreased TG and limited increase in oxidized glutathione levels (1.1 vs 12.9 μM; p = 0.04) (Table 3). However, the difference disappeared in the intent-to-treat analysis for oxidized glutathione but was maintained for TG (S1 Table).

**Table 3:** Change of variables of oxidative stress in HIV+ patients assigned to placebo or omega 3 fatty acids. Data are showed as mean (95% CI).

Variable	Omega 3 fatty acids	Placebo	p
Lipoperoxides (nM/mg protein)	-1.0 [(-)1.6 to 0.5]	-0.7 [(-)1.25 to (-)0.2]	0.36
Total glutathione (μM)	32.9 (9.1 to 56.6)	40.5 (20.8 to 60.2)	0.61
Oxidized glutathione (μM)	1.1 [(-)11.2 to 13.5]	12.9 (8.5 to 17.4)	0.04
Reduced glutathione (μM)	31.7 (12.8 to 50.5)	27.5 (8.5 to 46.5)	0.75
NO (μmoles/ml)	-21.1 ± 14.6	-22.6 ± 14.6	0.69
Triglycerides (mmol/L)	-0.32[(-)0.98 to 0.32]	0.54 [0.01 to 1.0]	0.04
Total cholesterol (mmol/L)	0.05 [(-)0.23 to 0.33]	0.08 [(-)0.17 to 0.34]	0.88
HDL cholesterol (mmol/L)	0.02 [(-)0.03 to 0.09]	0.02 [(-)0.04 to 0.10]	0.94
LDL-cholesterol (mmol/L)	0.05 [(-)0.19 to 0.31]	0.06 [(-)0.36 to 0.23]	0.52
Viral load (copies/mL)	-396 [-1170 to 377]	90.8 [-258 to 440]	0.29
CD4 count (cel/ml)	52 [(-)12 to 116]	-2.5 [(-)78 to 73]	0.26
CD8 count (cel/ml)	-184 [(-)427 to 58]	-227 [(-)353 to -102]	0.74
CD4/CD8	3.3 [2.7 to 4.0]	4.0 [3.1 to 4.9]	0.19
ALT (UI/L)	2.0 [(-)4 to 11]	0.0 [(-)3 to 14]	0.70
AST (UI/L)	1.0[(-)2 to 3]	0.0[(-)1 to 4]	0.82

No difference was found in change of viral load, CD4, CD8, CD4/CD8 ratio between groups at the end of treatment (Table 3).

No difference was found on adverse events between groups, diarrhea and dizziness was reported in patients assigned to fatty acids and biliary colic was related to placebo intake (S2 Table).

## Discussion

The World Health Organization considers that HIV/AIDS and CHD will be in the top 3 causes for both global mortality and global disability-adjusted life-years in the year 2030 [18]. The hypothesis of complex interplay of factors for cardiovascular disease is supported by the association of HIV with multiple vascular indices reflecting progressive stages of atherosclerosis, ranging from endothelial dysfunction [19] to coronary plaque itself [20]. No difference in viral load at the end of the study was found. However, in those who received omega 3 fatty acids, viral load trend to decrease while in the control group it trend to increase. Fatty acids inactivate animal enveloped viruses such as myxoviruses, paramyxoviruses, arboviruses, and herpes viruses within minutes of contact at a concentration of 5–25 μg/ml [21], and it has been considered that DHA may also possess similar capacity to inactivate HIV [22]. It is relevant because HIV infection in itself may cause detrimental changes in the vascular endothelium. For example, HIV viral load has been found to be associated with endothelial dysfunction [23] and HIV-1 Tat can promote the secretion of the chemokine MCP-1, thus favoring migration of monocytes into the vascular intima [24]. In addition, HIV Nef protein has been demonstrated to impair efflux of cholesterol from macrophages by downregulating adenosine triphosphate binding cassette transporter A1, and therefore increasing the promotion of foam cell formation [25].

In our study the effect of 2.4g omega 3 fatty acids on oxidative stress was evaluated. We found that in HIV-seropositive patients, this dose decreased triglycerides (TG) as has been previously reported in these patients [11]. Also Parandi et al [26] reported a decrease of 63.2 ± 86.9 mg/dl without change in total cholesterol, LDL-C, or HDL-C in 41 HIV+ subjects with hypertriglyceridemia (>150 mg/dl) after omega 3 fatty acid treatment (1.9 g EPA and 1.5 g DHA). However; Oliveira et al [27] found no effect on lipids after using 3 g fish oil in similar patients.

The relationship between circulating TG levels and atherosclerosis is still unclear. However, in some studies triglyceride has been identified as a “risk factor” in case-control and angiographic studies, even after adjustment for total cholesterol (TC), LDL-C and HDL-C [28–30]. Hypertriglyceridemia could be related with oxidative stress. For example, lipolysis of triglyceride-rich lipoproteins liberates free fatty acids, lysolecithin and a number of epoxides and oxidized lipids [31]. Also, many of these lipids reduced activity of endothelial nitric oxide synthase (eNOS) and increased production of reactive oxygen species (ROS) [32]. However, the reduction in TG levels was small. This could be secondary to TG levels in our patients, that were only close to borderline high and the effect of both EPA and DHA lowering TG has reported higher in cases of severe hypertriglyceridemia when TG levels exceed 5.5 mmol/l [33].

Previous trials in patients with different pathologies have demonstrated improved flow-mediated arterial dilation, a measure of endothelial function and health, after n-3 PUFA supplementation [34–37]. Because endothelial health is strongly linked to endothelial nitric oxide synthesis, experimental effects of n-3 PUFA on related biomarkers provide plausible biological mechanisms for such effects [38–39]. However, we did not find changes in nitric oxide levels between groups. This results are against studies that reported fatty acids enhances nitric oxide production by cultured human endothelial cells [40], Ca(2_)-independent activation and translocation of endothelial nitric oxide synthase and endothelium-dependent vasorelaxation in the rat [39]. This could be explained because our patients were normotensives and more studies are needed in vivo in humans.

We also found in the group that received omega 3 fatty acids a lower increase in oxidized glutathione levels than in those assigned to placebo in the per protocol analysis. However this difference disappeared in the intent-to-treat analysis (S1 Table). This variable is relevant because recently it has been described that perturbations in protein glutathionylation status, related with oxidized glutathione may contribute to the etiology of many cardiovascular diseases, such as myocardial infarction, cardiac hypertrophy and atherosclerosis. *S*-glutathionylation is a redox-dependent post-translational modification with growing relevance in signal transduction. Actually, *S*-glutathionylation is considered a regulatory event in “redox signaling”. The reversibility of this process is a key element to ascribing regulatory, as well as signaling functions to *S*-glutathionylation [41]. The deglutathionylation may occur via direct thiol/disulfide exchange reactions with GSH, once an appropriate GSH/GSSG ratio has been restored, or by the intervention of glutaredoxin [42].

In our study, near of 75% patients received two HAART therapy regimens. In HIV patients, non-traditional cardiovascular risk factors are low CD4 count, lipodystrophy syndrome, C hepatitis co-infection, metabolic syndrome, end-stage renal disease and antiretroviral therapy [43]. The D:A:D study (Data Collection in Adverse Effects of Anti-HIV Drugs) indicated that cumulative exposure to specific protease inhibitor (Lopinavir, Ritonavir and Indinavir) was associated with an increased risk of myocardial infarction (MI) [2]. There was no significant association between the development of MI an accumulative exposure to an NNRTI. For the NRTIs group, the only significant association between MI risk and cumulative exposure was with Abacavir, RR 1.07 [95% CI 1.09–1.82] or Didanosine, RR 1.41 [95% IC 1.09–1.82] [5]. However, other studies including a meta-analysis showed no significant association between Abacavir use and MI risk [44].

### Limitations of the study

Although sample size estimation was performed, an insufficient power due to the small sample size can be a reason for the lack of statistical difference in many of those parameters compared between the groups. Furthermore, in the intent to treat analysis we evaluated all patients included, even though they showed 50% or less adherence to treatment. Also, considering that HAART therapy could be a powerful factor that contributes to cardiovascular disease even the data continues to be contradictory; more studies are needed to evaluate the effect of omega 3 fatty acids on oxidative stress and CHD according to different therapy and in patients with different comorbidities. So it is necessary to confirm these results with a bigger sample size.

## Supporting Information

S1 CONSORT Checklist Consort 2010 checklist. (PDF)

S1 Table (DOC)

S2 Table (DOCX)

S1 Protocol (DOC)
