# Supplementary material for: Omega 3 Fatty Acids Supplementation and Oxidative Stress in HIV-Seropositive Patients. A Clinical Trial
Source: PLoS One. 2016 Mar 25;11(3):e0151637. doi: 10.1371/journal.pone.0151637 (PMC4807787; doi:10.1371/journal.pone.0151637)
Supplement: S1 Table — (DOC) [file pone.0151637.s002.doc]

S1 Table. Intent to treat analysis for change in triglycerides and oxidized glutathione levels in HIV+ patients receiving omega 3 fatty acids or placebo.

| Variable | Omega 3 fatty acids | Placebo | p |
| --- | --- | --- | --- |
| Triglycerides (mmol/L) | -0.31[(-)0.90 to 0.26] | 0.39 [(-)0.03 to 0.83] | 0.04 |
| Oxidized glutathione (μM) | 1.5 [(-)9.6 to 12.7] | 9.8 (5.9 to 13.8) | 0.15 |
