# Supplementary material for: Omega 3 Fatty Acids Supplementation and Oxidative Stress in HIV-Seropositive Patients. A Clinical Trial
Source: PLoS One. 2016 Mar 25;11(3):e0151637. doi: 10.1371/journal.pone.0151637 (PMC4807787; doi:10.1371/journal.pone.0151637)
Supplement: S2 Table — (DOCX) [file pone.0151637.s003.docx]

S2 Table. Adverse events in HIV+ patients receiving omega 3 fatty acids or placebo.

| Variable | Omega 3 fatty acids | Placebo | p |
| --- | --- | --- | --- |
|  |  |  | 0.54 |
| Gastrointestinal disorders |  |  |  |
| Biliar colic | - | 1 |  |
| Diarrhea | 1 | - |  |
| Nervous system disorders |  |  |  |
| Dizziness | 1 | - |  |
