# Supplementary material for: Omega 3 Fatty Acids Supplementation and Oxidative Stress in HIV-Seropositive Patients. A Clinical Trial
Source: PLoS One. 2016 Mar 25;11(3):e0151637. doi: 10.1371/journal.pone.0151637 (PMC4807787; doi:10.1371/journal.pone.0151637)
Supplement: S1 Protocol — (DOC) [file pone.0151637.s004.doc]

# INSTITUTO MEXICANO DEL SEGURO SOCIAL

#### DELEGACIÓN ESTATAL EN GUANAJUATO

#### UNIDAD MÉDICA DE ALTA ESPECIALIDAD NO. 1 BAJÍO

**EFfect of**

**omega 3 acids ON OXIDATIVE STRESS in seropositive HIV patients**

**Abstract**

**Antecedents**: highly active antiretroviral therapy (HAART) has showed its effectiveness in the prevention of complications in seropositive HIV patients. However, they develop some manifestations such as lipodystrophy, dyslipidemia, and glucose intolerance increasing cardiovascular risk.

Epidemiological evidence on the benefits of fish oil and omega 3 fatty acid consumption has been accumulated over the past few decades. Clinical trials with fish oil have demonstrated a significant reduction in cardiovascular events. Omega-3 fatty acids are believed to be beneficial in prevention of atherosclerosis reducing lipids levels specially triglycerides in general populations and in patients on hemodialysis. Also in general populations it has been described a benefic effect of omega 3 acids on oxidative stress.

**Objetive**: to know the effect of omega 3 acids on different markers of oxidative stress in seropositive HIV patients.

**Methods**: We will perform a randomized parallel controlled clinical trial in seropositive HIV patients from 20 to 55 years old on clinical score A1, A2, B1 or B2 who received HAART. Exclusion criteria will be diabetes mellitus, hypertension, use of hypolipidemic agents, dyslipidemia identified before receiving HAART therapy, and use of protease inhibitors. They will be randomly assigned to receive omega 3 fatty acids 2.4 g (Zonelabs, Marblehead MA) or placebo for 6 months. At baseline anthropometric measurements will be performed. After at least 8 fasting hours a venous 20 ml blood sample of will be obtained to measure lipid profile, glucose and stress oxidative levels (nitric oxide, malondialdehyde, total glutathione and lipid peroxidation products).

Sample size was calculated according to different variables. For example for malondialdehyde we consider a previous study in patients on hemodialysis (22) where after 2 months of omega 3 fatty acids treatment, a difference of 0.9 nmol/l was found between groups, with a SD=0.9 and 0.7 nmol/l in the treatment group and in the control group respectively. Whit this information we obtained a sample size of 17 patients per group for an 80% statistical power with α= 0.05. Similar sample size was calculated for glutathione considering a difference=500 µg/l between groups and SD=700 µg/l (48). Whit this information we obtained a sample size of 31 patients per group for an 80% statistical power with α= 0.05. For nitric oxide we calculated a sample size of 30 patients per group considering a difference in 25% in its levels between groups and SD=10µmol/L with similar statistical power and α value (49). Assuming a 15% patient lost, a sample size of 35 per group was considered.

**Resources and infrastructure*:*** In our hospital, enough number of HIV+ patients receives medical attention to reach the sample size in a mean period of a year. All clinical determinations are factible to perform in the CIBO in Jalisco state. We will compete for funding to obtain laboratory reagents and drugs.

**Experience of the group**: The working group is composed of researchers with experience in the pharmacological management and complications of these patients and in research methodology. Several publications of some researchers have proven oxidative stress in various pathologies.

**Time to develop**: Once registered with the National Commission of Research, we will compete for resources and will start the protocol to complete the sample size and follow-up of patients.

**Antecedents**

In Mexico, as in other countries, the Acquired Immune Deficiency Syndrome (AIDS) has become a very complex priority public health problem that threatens national security and for economic and social development nations. Mexico is classified as a country with a concentrated epidemic of AIDS, characterized by a prevalence of HIV infection has spread rapidly in one or more subsets of the population, but is not yet established in the general population. In this type of epidemics, HIV prevalence has remained constant over 5% in at least one subgroup of the population, and among pregnant women in urban areas is less than 1% (1).

The AIDS epidemic in Mexico is concentrated in some populations at higher risk, such as sex workers (men) with a mean prevalence of 15%. Then men who have sex with men (11%), injection drug users (5%), sex workers (2%) and persons deprived of liberty (1%). Clients of sex workers and heterosexual men and women report significantly lower prevalence. According to estimates by the National Center for the Prevention and Control of AIDS (CENSIDA), together with UNAIDS, in Mexico there are 220,000 adults living with HIV (2009), of which 60% are men who you have sex with men (MSM), 23% of heterosexual women and 6% for clients of sex workers, primarily heterosexual (1). By 2010, the same source cites national 144.127 cumulative AIDS cases with an incidence of 133 patients per 100,000 inhabitants, being the most affected age groups of 15 to 44 years of male dominance (82.18%) (2).

Before the arrival in Mexico of antiretroviral treatment, the overall death rate from AIDS was 1.8 deaths per hundred thousand inhabitants in 1990 and peaked in 1996 with 4.8 deaths registration. From that last year, progressive access to a new group of antiretroviral drugs known as protease inhibitors (PI) radically changed the prognosis of patients with HIV / AIDS. It has been shown that the combination of drugs known as highly active antiretroviral therapy (HAART by its acronym in English), slows the damage of the immune system and substantially improve the quality of life (1).

However, these benefits have been overshadowed by the emergence of new side effects as effective treatment, including highlights its deleterious effect on lipoprotein metabolism. This is characterized by elevated levels of very low-density lipoprotein and triglycerides, elevated total cholesterol, low density lipoprotein and decreased levels of high density lipoprotein. Triant compared rates of myocardial infarction in two hospitals in Boston with more than 3,800 patients, and reported a significantly higher rate in HIV + compared to HIV negative, in addition to a higher prevalence of risk factors such as hypertension, diabetes mellitus and dyslipidemia (3).

HAART has shown its effectiveness in preventing complications of the disease and increase survival (4). Furthermore, it has been reported that 20% of patients undergoing HAART developed lipodystrophy after 12 months of treatment, 2% diabetes mellitus and 25% dyslipidemia (which was as high as 41% in case of low HDL-cholesterol) at 15 months of treatment (5). Lipodystrophy is characterized by changes in the distribution of body fat (fat accumulation in the central region of the body especially the trunk, abdomen and dorsocervical peripheral region accompanied by atrophy of subcutaneous fat, dyslipidemia and insulin resistance) (6 7), which also increases the risk of cardiovascular disease by participating in the process of atherosclerosis (8).

Atherosclerosis is a chronic inflammation syndrome with predilection on artery walls. In the initial phase of development of this syndrome, low density lipoprotein (LDL) and other pro-inflammatory proteins stimulate the expression of adhesion factors of endothelial cells and growth factors that attract circulating monocytes to endothelial cells penetrating the intimate where colony stimulating factors induce macrophage differentiation of monocyte-macrophage. Macrophage uptake of oxidized LDL cholesterol promotes their further evolution into foam cells, important players in atherosclerosis development. Then, activation of T cells, proliferation of smooth muscle cells and deposition of extracellular matrix are produced in the vascular intima. These molecules interact with deposits in the intima producing necrosis, causing the formation of fibrous plaques containing a core of lipid and a fibrous layer formed of smooth muscle cells and matrix. Eventually the progressive growth of plaque rupture can reach the blood stream and cause stenosis or closure of the lumen of other arteries. Furthermore, as a result of plaque rupture, platelet activation generates thrombosis obstruction of blood flow resulting in acute myocardial ischemia or cerebral vascular disease. Then, atherosclerosis is a dynamic process and one of its main triggers is oxidative stress (9).

**Oxidative stress**

Oxidative stress is defined as a situation where there is an increase in the rate of generation of oxidative species and free radicals (FR) and a decrease in the activity of the defense systems, resulting in higher concentrations at steady state active of oxygen species. In these situations, the toxic effects of FR result in chemical reactions on lipids, proteins, carbohydrates and DNA, which may result in irreversible damage and even cell death (10).

There are numerous diseases that have been associated with this imbalance between oxidants and antioxidants: atherosclerosis, cancer, porphyria, cataracts, iron and copper overload, Parkinson's disease, Alzheimer's disease, diabetes, etc. (10).

One of the biggest contributors of oxidative stress is the family of molecules reactive oxygen species (ROS). These include free radicals such as superoxide anion (O2-), hydroxyl radical (HO-), lipid radicals (ROO) and nitric oxide (NO). Other reactive oxygen species, such as hydrogen peroxide (H2O2), peroxynitrite (ONOO-) and hypochlorous acid (HOCl), although they are not free radicals, have oxidizers effects that contribute to oxidative stress. ROS have been implicated in cellular necrosis and apoptosis damage due to its direct oxidizing effects on macromolecules such as lipids, proteins and DNA (11-12). Reactions between FR and polyunsaturated fats in the cell membrane can result in peroxyl acid radicals that accumulates in the cell membrane disrupting protein function and signal transduction. Under oxidative stress, the superoxide ion release free iron of various molecules to produce highly reactive hydroxyl radicals by the Fenton reaction with hydrogen peroxide (13). ROS also induce the opening of the permeability transition pore mitochondrial membrane and release of cytochrome C and other factors that may cause apoptosis (14-15). The O2- radicals can also interact with nitric oxide (NO) signaling, resulting in the formation of reactive nitrogen species (RNS) that reduce the bioavailability of NO causing "nitrosative stress" toxicity (16). Excessive production of RNS results in nitrosylation reactions changing the structure of proteins with loss or change of their functions (17).

Moreover, it has been shown that increased oxidative stress is attached to the HIV infection. Canadian researchers suggested that a weak antioxidant defense system is a significant factor when considering the high oxidative stress observed in the population infected by the virus*.* The increased production of reactive oxygen molecules might stimulate the replication of HIV and related immunodeficiency (18).

ROS are a staple in antimicrobial defense and antitumor. Oxidative stress is recognized associated with the pathophysiology of several diseases. Experimental evidence links the process of oxidative stress in the development of viral diseases such as infection by human immunodeficiency virus (HIV) (19).

Under physiological conditions the cells increase their activity of antioxidant enzymes and other defenses to balance oxidative stress (20-21). These include manganese dependent superoxide dismutase (Mn-SOD), superoxide dismutase copper / zinc (Cu / Zn SOD), glutathione peroxidase, glutathione reductase and catalase (CAT). Other antioxidant defenses include "scavengers" of free radicals as vitamin E, beta carotene and vitamin C (EO4) and recently the participation of omega 3 fatty acids has been also considered as antioxidants (22).

**Oxidative stress in HIV+ patients**

The first evidence of the presence of EO in these individuals was reported in 1988 with an increase of malondialdehyde (MDA) as an index of oxidative damage in HIV-positive patients compared with a group of healthy individuals, and in 1989 in HIV-positive individuals, deficiency of systemic glutathione (GSH) was reported (19).

The relationship between infection with human immunodeficiency virus (HIV), nutrition and immune function is complex. The effect of HIV infection on nutritional status is unclear; however the effect of nutritional status on the infection has not been adequately evaluated (23).

The OE has been considered a cofactor in the progression of HIV infection to AIDS condition, associated with an increase in viral load and the decrease in CD4 + T cells (apoptosis) producing a depression in the immune response (19).

**Omega 3 fatty acids**

Omega 3 fatty acids are essential fatty acids, because the body cannot manufacture them from other substances and belong to the group of polyunsaturated fatty acids. It is estimated that the ideal ratio between these two types of fatty acids to include in our diet could be 4 parts omega 3 and 1 part omega-6 fatty acids.

Recently in JELIS (24) study that followed-up patients for five years, it was reported that those Japanese patients with dyslipidemia who received 1800 mg of eicosapentaenoic acid (C20: 5n-3, EPA) per day alone or in combination with statins, reduced event rate of major cardiovascular disease. Also the results of the study "GISSI Prevenzione" reported reduced risk of sudden death from 4 months post-infarction with the use of EPA and docosahexaenoic acid (C22: 6n-3, DHA), and this effect remained in the 3.5 years of follow-up (25). Other mechanisms proposed by lowering cardiovascular risk in people with dyslipidemia are their hypotensive effect, (26) vascular relaxation, (27) and increased heart rate variability (28). In hypertensive rats, DHA treatment for 6 weeks reduced the vascular wall thickness of the coronary artery and aorta (29). Also in humans, atherosclerotic plaques of patients undergoing carotid endarterectomy, who received Ώ-3 for ~42 days showed less macrophages infiltration compared with control group and were more resistant to rupture (30). The European and American cardiology societies have included EPA and DHA in the new treatment guidelines for myocardial infarction (31).

Ώ-3 fatty acids ‘doses for multiple outcomes varied between studies. For example, Freeman reported decrease in the level of postpartum depression after 8 weeks with 2.8 g per day of EPA + DHA (32). Geelen et al. (33) demonstrated that 14 weeks of a moderate dose (1,260 mg / day) reduced the heart rate in patients with complex ventricular arrhythmias and based on the GISSI-HF study (34) for heart failure has been established recommendation of use 800-1,000 mg / day of EPA / DHA in patients at high cardiovascular risk (34-35). Moreover, in patients with hypertriglyceridemia it has been reported that it is safe to administer even dose 4g / day of Ώ-3 fatty acids combined with other lipid-lowering therapies such as statins, fibrates and niacin (36-39). Even the American Heart Association supports the dose of 1g / day of EPA and DHA in combination in patients with documented cardiovascular disease (40). Because adverse effects have been reported more frequently in doses from 4 g and its effectiveness is demonstrated on average from 1 g, we decided to study an intermediate dose of 2.4 g (41-42).

Although details of the cellular mechanism by which these acids lower serum triglycerides are unknown, EPA and DHA can reduce VLDL synthesis (43), increased triglyceride clearance of VLDL (44) and increase the conversion remnants of VLDL to LDL (45). In a recent study in patients undergoing hemodialysis, supplementation with omega-3 increased levels of antioxidants factors such as glutathione peroxidase and superoxide dismutase after two months of treatment, and decreased levels of malondialdehyde, improving oxidation state (22).

The safety and efficacy of omega-3 fatty acids for the treatment of hypertriglyceridemia in the general population is well documented (45-46). In addition to the "fishy taste", adverse effects are alterations in intestinal transit, and it has been reported that reduces the generation of thrombin independently of vitamin K (41), but not increases bleeding risk (42). Even in patients receiving other coumarin anticoagulants and antiplatelet agents, has not been observed increase in the rate of bleeding episodes that required decrease anticoagulant dosage (47).

**Justification**

An antioxidant therapy in combination with antiretrovirals may benefit HIV-infected patients, extending and improving their quality of life. This could significantly reduce oxidative stress which is a trigger for several related diseases such as diabetes mellitus, hypertension and cardiovascular disease in general.

The use of omega-3 as complementary treatment opens the possibility to reduce cardiovascular risk by reduction of oxidative stress in these patients without drug interactions observed with the use of statins.

**Problem identification**

What is the effect of using omega-3s on various markers of oxidative stress in HIV+ patients?

**PRIMARY OBJECTIVE:** to know the effect of omega 3 acids on different markers of oxidative stress in HIV+ patients.

Change on malondialdehyde, nitric oxide and glutathione levels after treatment with omega 3 acids for 6 months compared with placebo

**Ha**: use for 6 months of omega 3 fatty acids, reduce oxidative stress in HIV+ patients.

**Ho**: use for 6 months of omega 3 fatty acids; do not reduce oxidative stress in HIV+ patients.

**Material and methods**

**Type of study**

Randomized, parallel, controlled clinical trial, doble blind.

**Universe of the study**

HIV-positive patients attending the AIDS Medical Unit of High Specialty Bajio, IMSS, in the service of Infectious Diseases, in the city of Leon, Guanajuato, Mexico.

**Sample size**

It was calculated according to different variables. For example, for malondialdehyde we consider a previous study in patients on hemodialysis (22) where after 2 months of omega 3 fatty acids treatment, a difference of 0.9 nmol/l was found between groups, with a SD=0.9 and 0.7 nmol/l in the treatment group and in the control group, respectively. With this information we obtained a sample size of 17 patients per group for a 80% statistical power with α= 0.05. Similar sample size was calculated for glutathione considering a difference=500 µg/l between groups and SD=700 µg/l (48). With this information we obtained a sample size of 31 patients per group for a 80% statistical power with α= 0.05. For nitric oxide we calculated a sample size of 30 patients per group considering a difference in 25% in its levels between groups and SD=10µmol/L with similar statistical power and α value (49). Assuming a 15% patient lost, a sample size of 35 per group was considered by t test for sample size (50).

**Sampling**

Sampling shall be consecutive cases who met the inclusion criteria, up to the given sample size. Patients will be randomly assigned to each group of treatment.

**Inclusion criteria: patients with**

HIV+ patients from 20 to 55 years old

On clinical score A1, A2, B1 or B2

Patients who received highly active antiretroviral therapy

Exclusion criteria

patients with diagnoses of diabetes mellitus, hypertension, use of hypolipidemic agents, dyslipidemia identified before receiving HAART therapy, and use of protease inhibitors.

- *At least one of the following alterations in lipids: Triglycerides between 200 to 500 mg/dL, LDL cholesterol between 130-160 mg/dL without use of hypolipidemic agents.
- CD4> 200 cel/mL
- Without anticoagulants use
- Patients non coinfected (B or C hepatitis, tuberculosis, etc.).

***** We decided these lipid values according to the “Executive summary of the third report of the National Cholesterol Education Program (NCEP) Expert Panel on Detection, Evaluation, and Treatment of High Blood Cholesterol in Adults (Adult Treatment Panel III)”, for which atherogenesis is significant (52).

**Exclusion criteria**

- All those patients who evolve to more advance clinical scores during the following in the study.
- In case of resistance to antiretroviral treatment they will be statistical analized.

**Material and methods:**

## ***In the department of*** ***infectious diseases*** ***of*** ***our hospital***, patients who ***met*** ***the inclusion criteria*** will be ***informed*** ***broadly*** ***the study's purpose***, procedures, risks and benefits. ***If*** ***accepted for participation***, after ***clarification*** ***of all*** ***doubts***, ***they will sign the*** ***informed*** ***consent letter*** ***(Appendix 1***). We will review all ***clinical*** ***records to determine the characteristics*** ***of the disease***, the time from ***the*** ***detection*** ***of the disease*** ***and the presence*** ***of complications or*** ***comorbidities***.

In any case we will select patients whose physicians’ using the clinical practice guideline IMSS 245-09 for use of antiretroviral drugs in adult patients with HIV infection (53). When a patient during follow up shows viral load> 1000 copies, sequence genotype will be requested and optimized treatment will be assigned if required.

Once corroborate that patients meet the inclusion criteria and have agreed to participate, they will be randomly assigned to either of the two treatment groups: omega 3 fatty acids, 2.4 g per day or placebo (olive oil gelcaps, Perfect Source, Fullerton CA, product code number PER 1016, lot number 8A0019/1600-1) in similar presentation as omega 3 fatty acids (Zonelabs, Marblehead MA), requiring intake 2 capsules in the morning and two at night. On this visit as basal stage, general determinations will be made about nutritional status according to food intake assessment (through the Food Processor). Anthropometric measurements will be performed as weight, height, BMI, waist and hip circumference.

For weight, a calibrated electronic scale portable analog Tanita will be used. It has a capacity of 120 kg and a precision level of ± 100 g. Patients will be weighed using under clothing. The waist and hip measurement will be done with a tape measure, fiberglass, flexible, inelastic 200cm and with an accuracy of ± 1 mm in accordance with the guidelines established (55).

For the size we will verify that the floor and wall are flat and stadimeter properly placed, the patient to take their shoes off and in case of women we will ask to avoid high hairstyles. We will check posture: feet together, heels touching the wall, hanging on the sides and looking straight ahead and finally we will obtain the data.

With the weight and height of patients we will obtain BMI. The waist and hip measurement will be done with a flexible tape, fiberglass, 200cm inelastic with an accuracy of ± 1 mm in accordance with the guidelines established (55).

All measurements will be performed before calibration of instruments, and will take place in both groups under similar conditions at the beginning and end of the study (6 months).

At visits 1, 2 and 4 or 5, after fasting for 12 hours, we will obtain a blood sample and determine the lipid profile (total cholesterol, triglycerides, LDL, HDL, VLDL), ALT and AST. At visit 2 we will perform lipid profile. Oxidative stress parameters will be determined only at baseline and final stage. For the latter purpose lipid peroxidation products, total glutathione levels and nitric oxide will be measured. When lipid profile revealed the persistence of basal levels or increase in both triglycerides and LDL-cholesterol, statins or fibrates will be added for hypercholesterolemia or hypertriglyceridemia respectively according to the "Executive summary of the third report of the National Cholesterol Education Program (NCEP) Expert Panel on Detection, Evaluation, and Treatment of High Blood Cholesterol in Adults (Adult Treatment Panel III) " (52).

1. **Lipid peroxidation**:

The production of malondialdehyde (MDA) and 4-hidroxialquenos (4-HDA) will be used as a marker of oxidative damage, for this reason test kit (Kit) LPO-586 will be used. This commercial kit has advantages over others since chromogen agent reacts with lipid peroxidation products: MDA and 4-HDA to 45^o^C, this reaction results in a stable product which has a maximum absorbance at 585 nM. The wavelength, and the low temperature incubation used eliminates undesirable artifacts. Proteins are quantified with the method of Bradford as following:

1. Sera will be homogenized on ice with a buffer of 20 nM Tris hydroxymethyl aminomethane at pH 7.4 with a Polytron (ultrasonic homogenizer) to produce a mixture 1:10, this homogenate will be centrifuged at 3,000 g for 30 minutes at 4°C. The supernatant will be collected and the lipid peroxidation products will be quantified: MDA and 4HDA.
2. **Determination of nitric oxide (nitrates and nitrites):**
3. Nitric oxide will be indirectly quantified because during their metabolism nitrates and nitrites are formed. We will use a commercial kit (Calbiochem Nitric Oxide Assay Kit, colorimetric 482650) which uses the nitrate reductase enzyme to convert the nitrates to nitrites, and subsequently through the Griess reagent nitrites are quantified.
4. The nitrates will be converted to nitrites by nitrate reductase incubation in the presence of NADPH, and then lactate dehydrogenase will be used to destroy NADPH. Equal volumes of sample and Griess reagent (V: V) will be incubated at room temperature (10 minutes). The absorbance will be measured at 550nm. Eventually the concentration of nitric oxide (nitrates / nitrites) will be determined by a curve of sodium nitrate as standard.
5. **Determination of total glutathione (GSH and GSSG):**
6. Total glutathione levels will be determined using the method of Griffith (56). The total glutathione is evaluated by the procedure wherein enzymatic recycling, GSH is oxidized by acid 5, 5'-dithiobis-2-nitrobenzoic acid (DTNB) and reduced by NADPH in the presence of glutathione reductase. The formation of 2-nitro-5-thiobenzoic (TNB) is monitored at 412 nm. The total glutathione in the sample is determined by comparing the observed value with a standard curve generated from known concentrations of GSH. GSSG sample will be determined by the above recycling method. The samples will be treated with 4-vinylpyridine. The 4-vinylpyridine is used in all samples to eliminate reduced glutathione, leaving only the oxidized form of glutathione as single test substrate (Suzuki, 1993). GSH will be calculated by subtracting the total glutathione GSSG.

## ***We will also measure*** ***levels*** ***of systemic arterial*** ***pressure*** ***with the patient*** ***in a sitting position*** ***prior*** ***rest*** ***at least 15 minutes***. ***We will place*** ***the cuff on the*** ***nondominant arm*** ***which*** ***will breathe*** ***with*** ***a mercury sphygmomanometer***. We will carry out ***at least two determinations*** ***with a minimum*** ***difference between them*** ***of*** ***five minutes*** ***and the average*** ***recorded. For*** ***nutritional assessment***, ***we explain*** ***the methodology of the*** ***24-hour recall*** ***and*** ***food diary*** ***for*** ***three days*** ***[***prior ***standardization*** ***by*** ***researchers***] ***(***58) to measure ***the average consumption of*** ***energy and*** ***macronutrients*** ***(proteins***, lipids, carbohydrates ***and fiber)*** ***to be analyzed*** ***by*** ***software*** ***FOOD*** ***PROCESSOR*** ***SQL*** ***baseline*** ***and 6-month*** ***phase*** ***where*** ***we will also measure*** ***the daily intake of*** ***omega*** ***3 and omega 6 fatty acids*** (59).

**Administration of omega 3 fatty acis**

We will indicate to patients in the group assigned to omega-3 (Zonelabs, Marblehead MA) to take the study drug twice daily with 150 ml of water, the first during or after breakfast and the second during or after dinner. The placebo will be an inert substance in a presentation similar to that of omega-3 acids and will be provided in both patients in a flask with similar presentation.

**Adherence**

The researcher will calculate tablets and compliance with treatment in a worksheet. Adherence to treatment will be calculated according to the following formula:

Adherence to treatment (%) = number of capsules actually taken from the last count / number of tablets should be taken at the same stage x 100%.

Adherence to treatment throughout the study protocol should be 80% to 120%. If it is outside this range, the patient will be carefully interviewed and told to report back to the purpose of the study and the possible effects expected.

**Statistical analysis**

The Mann–Whitney *U* test or unpaired Student’s *t-*test will be performed to evaluate differences between baseline and final variables in group assigned to omega-3 fatty acids or placebo for variables displaying no normal or normal distribution, respectively. Chi-square test was used for categorical variables. A *p* value < 0.05 will be considered significant. All data will be analyzed using the Statistics software version 6.0 (Statsoft Inc., Tulsa, OK, USA).

**Ethical considerations**

The proposed procedures are in accordance with ethical standards, the Regulations of the General Law of Health in Research for Health and the Helsinki declaration of 1975 as amended, as well as codes and national and international standards for good practice in research. The risk of research is considered greater than the minimum but with prospect of direct benefit to each of the participants. However the side effects have been reported with the use of omega as alterations in the intestinal transit (diarrhea), decrease in blood pressure and prolonged bleeding times (letter of informed consent) are also specified. Possible contributions and benefits of the study is to identify a therapy decreases oxidative stress in HIV seropositive patients and long-term decrease cardiovascular risk by improving the survival and quality of life of these patients. The proposed drug is not part of the core box at the institution but is considered likely to be increased evidence of its effectiveness in several diseases could be included in the medium term.

**Privacy and confidentiality.** Patient information that could be used for identification (such as name, address or telephone number) will be kept confidential and separate as well as their responses to the questionnaires and the results of clinical tests, to ensure their privacy and no one else outside the research team (including the physician) will have access to information that the patients provides for their participation in this study. If the results of this study are published or presented at conferences, it will not be given information that might reveal the identity of patients.

Informed consent will be requested by two of the investigators (NAL and SCMM) that are not part of medical care for HIV seropositive patients, once a review of records of outpatients who meet inclusion criteria and later identified a comprehensive explanation of the purpose of the research project, its risks and expected benefits. The selection of participants will be by consecutive cases according to inclusion criteria, acceptance of their participation until complete the sample size. According to hospital records we consider that a third of patients that are currently part of the census of the HIV clinic (approximately 180 patients) would be potential participants.

In any case it will be explained to the patient the purpose and risks of their participation in the study and signed the informed along with two witnesses consent and will emphasize that he can withdraw from the study when deemed without affecting your care of the institution.

**References**

1. Centro Nacional para la Prevención y Control del SIDA (CENSIDA). El VIH/SIDA en México 2009.

2. Centro Nacional para la Prevención y Control del SIDA (CENSIDA) 2010. Disponible en [http://www.censida.salud.gob.mx](http://www.censida.salud.gob.mx/) Revisado el 25 de marzo del 2011.

3. Triant VA, Lee H, Hadigan C, Grinspoon SK. Increased acute myocardial infarction rates and cardiovascular risk factors among patients with human immunodeficiency virus didease. J Clin Endocrinol Metabol 2007; 92: 2506-2512.

4.- Yeni P. Update on HAART in HIV. J Hepatol 2006;44:100–103.

5. Carr A, Samaras K, Thorisdottir A. Diagnosis, prediction, and natural course of HIV-1 protease-inhibitor-associated lipodystrophy, hyperlipidaemia, and diabetes mellitus: a cohort study. Lancet 1999;353:2093–2099.

6.- Galli M, Ridolfo AL, Gervasoni C. Cardiovascular disease risk factors in HIV-infected patients in the HAART era. Ann N Y Acad Sci 2001;946:200–213.

7.-Koutkia P, Grinspoon S. HIV-associated lipodystrophy: pathogenesis, prognosis, treatment, and controversies. Annu Rev Med 2004;55:303–317.

8.- Kamin DS, Grinspoon SK. Cardiovascular disease in HIV-positive patients. AIDS 2005;19:641–652.

9. Galis ZS, Khatri JJ. Matrix metalloproteinases in vascular remodeling and atherogenesis: the good, the bad, and the ugly. *Circ Res* 2002; **90**: 251-262

10.- Feher J, Csomos GA. Free radical reactions in medicine. Vol 42. New York: Springer Verlag; 1987:71-79

11. Valko M, Rhodes CJ, Moncol J, Izakovic M, Mazur M. Free radicals, metals and antioxidants in oxidative stress-induced cancer. Chem Biol Interact 2006;

160:1-40.

12. Valko M, Morris H, Cronin MT. Metals, toxicity and oxidative stress. Curr Med Chem 2005; 12:1161-208.

13. Jang S, Imlay JA. Micromolar intracellular hydrogen peroxide disrupts metabolism by damaging ironsulfur enzymes. J Biol Chem 2007; 282:929-37.

14. Tatton W, Chalmers-Redman R, Tatton N. Neuroprotection by deprenyl and other propargylamines: glyceraldehyde-3-phosphate dehydrogenase rather than monoamine oxidase B. J Neural Transm 2003; 110:509-15.

15. Tsutsui H, Kinugawa S, Matsushima S. Mitochondrial oxidative stress and dysfunction in myocardial remodelling. Cardiovasc Res 2009; 81:449-56.

16. Elahi MM, Naseem KM, Matata BM. Nitric oxide in blood. The nitrosative-oxidative disequilibrium hypothesis on the pathogenesis of cardiovascular

disease. Febs J 2007; 274:906-23.

17. Ridnour LA, Thomas DD, Mancardi D, Espey MG, Miranda KM, Paolocci N, et al. The chemistry of nitrosative stress induced by nitric oxide and reactive nitrogen oxide species. Putting perspective on stressful biological situations. Biol Chem 2004; 385:1-10.

18. Allard JP, Aghdassi E, Chau J, Salit I, Walmsley S. Oxidative stress and plasma antioxidant micronutrients in humans with HIV infection. Am J Clin Nutr 1998;67:143-147.

19. Gil del Valle L, Reyes AT, Sánchez GM, Fernández OSL. Terapia antioxidante en la infección por el virus de la inmunodeficiencia humana. Acta Farm Bonaerense 2002; 21:301-308.

20. Dröge W. Free radicals in the physiological control of cell function. Physiol Rev 2002; 82:47-95.

21. Vassalle C, Pratali L, Boni C, Mercuri A, Ndreu R. An oxidative stress score as a combined measure of the pro-oxidant and anti-oxidant counterparts in patients with coronary artery disease. Clin Biochem 2008; 41:1162-7.

22. Tayyebi-Khosroshahi H, Houshyar J, Tabrizi A, Vatankhah AM, Razzagi Zonouz N, Dehghan-Hesari R. Effect of omega-3 fatty acids in oxidative stress in patients on hemodialysis. Iran J Kidney Dis 2010; 4:322-326.

23.- De Luis DA, Bachiller P, Izaola O, Eiros Bouza JM. Relation between intake of omega 3 fatty acids and CD4 count in patients with HIV infection: a transversal study. An Med Interna 2005; 22:323-325.

24.- Yokoyama M, Origasa H, Matsuzaki M, Matsuzawa Y, Saito Y, Ishikawa Y, et al. Japan EPA lipid intervention study (JELIS) Investigators. Effects of eicosapentaenoic acid on major coronary events in hypercholesterolemia patients: A randomized open-label, blinded endpoint analysis. Lancet 2007;369:1090-1098.

25.- Marchioli R, Barzi F, Bomba E, Chieffo C, Di Gregorio D, Di Mascio E. et al. Valagussa on behalf of the GISSI-Prevenzione Investigators. Early protection against sudden death by n-3 polyunsaturated fatty acids after myocardial infarction. Time course analysis of the results of the Gruppo Italiano per lo Studio della sopravvivenza nell Infarto Miocardico (GISSI)-Prevenzione. Circulation 2002;105:1897-1903.

26.- Geleijnse JM, Giltay EJ, Grobbee DE, Donders AR, Kok FJ. Blood pressure response to fish oil supplementation: metaregression analysis of randomized trials. J Hypertens 2002;20:1493-1499.

27.- Goodfellow J, Bellamy MF, Ramsey MW, Jones CJ, Lewis MJ. Dietary supplementation with marine omega-3 fatty acids improve systemic large artery endothelial function in subjects with hypercholesterolemia. J Am Coll Cardiol 2000;35:265-270.

28.- Calder PC. n-3 Fatty acids and cardiovascular disease: evidence explained and mechanisms explored. Clin Sci 2004;107:1-11.

29.- Engler MM, Engler MB, Goodfriend TL, et al. Effects of docosahexaenoic acid on vascular pathology and reactivity in hypertension. Exp Biol Med 2003;228:229-307.

30.- Thies F, Garry JM, Yaqoob P, et al. Association of n-3 polyunsaturated fatty acids with stability of atherosclerotic plaques: a randomized controlled trial. Lancet 2003;361:477-85.

31.- Von Schacky C, Angerer P, Kothny W, Theisen K, Mudra H. Omega-3 fatty acids and cardiovascular disease. Curr Opin Clin Nutr Metab Care 2007;10:129-135.

32. Freeman MP, Hibbeln JR, Wisner KL, Brumbach BH, Watchman M, Gelenberg AJ. Randomized

dose-ranging pilot trial of omega-3 fatty acids for postpartum depression. Acta Psychiatr Scand. 2006; 113:31–35.

33. Geelen A, Brouwer IA, Schouten EG, Maan AC, Katan MB, Zock PI. Effects of n-3 fatty acids from fish on premature ventricular complexes and heart rate in humans. Am J Clin Nutr 2005;81:416 –20.

34. GISSI-HF Investigators. Effect of n-3 polyunsaturated fatty acids in patients with chronic heart failure (the GISSI-HF trial): a randomised, double-blind, placebo-controlled trial. Lancet 2008;372:1223–30.

35. Fonarow GC. Statins and n-3 fatty acid supplementation in heart failure. Lancet 2008;372:1195– 6.

36. Bays H. Clinical overview of Omacor: a concentrated formulation of omega-3 polyunsaturated fatty acids. Am J Cardiol 2006;98:71i– 6i.

37. Bays H. Rationale for prescription omega-3-acid ethylester therapy for

hypertriglyceridemia: a primer for clinicians. Drugs Today (Barc) 2008;44:205– 46.

38. Bays H, Tighe AP, Sadovsky R, Davidson MH. Prescription omega-3 fatty acids and their lipid effects: physiologic mechanisms of action and clinical implications. Expert Rev Cardiovasc Ther 2008;6:391– 409.

39. Harris WS, Jacobson TA. Omega-3 fatty acids. In: Ballantyne CM, editor. Clinical Lipidology: A Companion to Braunwald’s Heart Disease. Philadelphia, PA: Saunders, an imprint of Elsevier Inc., 2009:326 –38.

40. Kris-Etherton PM, Harris WS, Appel LJ, for the American Heart Association Nutrition Committee. Fish consumption, fish oil, omega-3 fatty acids, and cardiovascular disease [published correction appears in Circulation 2003;107:512]. Circulation 2002;106:2747–57.

41.- 36.- Vanschoonbeek K, Feijge M, Paquay M, et al. Variable hypocoagulant effect of fish oil intake in humans: modulation of fibrinogen level and thrombin generation. Arterioscler Thromb Vasc Biol 2004;24:1734-1740.

42.- 37.- Bays HE. Safety considerations with omega-3 fatty acid therapy. Am J Cardiol 2007;99:35C-43C.

43.- Harris WS, Bulchandani D. Why do omega-3 fatty acids lower serum tryglicerides? Curr Opin Lipidol 2006;17:387-393.

44.- Park Y, Harris WS. Omega-3 fatty acid supplementation accelerates chylomicron triglyceride clearance. J Lipid Res 2003;44:455-463.

45.- Durrington P, Bhatnagar D, Mackness M et al. An omega-3 polyunsaturated fatty acid concentrate administered for one year decreased triglycerides in simvastatin treated patients with coronary heart disease and persisting hypertrygliceridemia. Heart 2001;85:544-548.

46.- Lewis A, Lookinland S, Beckstrand R, Tiedeman M. Treatment of hypertriglyceridemia with omega-3 fatty acids: a systematic review. J Am Acad Nurse Pract 2004;16:384-395.

47.- Bender N, Kraynak M, Chiquette E, et al. Effects of marine fish oil on the anticoagulation status of patients receiving chronic warfarin therapy. J Thromb Thrombolysis 1998;5:257-261.

48. Flagg EW, Coatesi RJ, Jones DP, Eley W, Gunter EW, Jackson B, Greenberg RS. Plasma total glutathione in humans and its association with demographic and health-related factors. B J Nutr 1993; 70:797-808.

49. Jia L, Yan-Ming S, Lan-Feng W, Zhu-Qin L, Wei P, Hong-Yan C. Comparison of effects of simvastatin versus atorvastatin on oxidative stress in patients with coronary heart disease. Clin Cardiol 2010; 33:222-227.

50. Browner WS, Black D, Newman T, Hulley SB. Estimación del tamaño de la muestra y de la potencia, in: Hulley SB, Cummings SR (Eds.). Diseño de la investigación clínica, DOYMA, Barcelona, España, 1993, pp. 153-165.

51.- Clasificación para la infección por VIH/SIDA. URL: <http://vihsida.org/?id=14>.

52.- Expert Panel on Detection, Evaluation and Treatment of High Blood Cholesterol in Adults. Executive summary of the third report of the National Cholesterol Education Program (NCEP) Expert Panel on Detection, Evaluation, and Treatment of High Blood Cholesterol in Adults (Adult Treatment Panel III). JAMA. 2001;285:2486-2497.

53. Guía de práctica clínica IMSS-245-09 para uso de antirretrovirales en pacientes adultos con infección por el VIH del Instituto Mexicano del Seguro Social. Disponible en [http://imss.gob.mx](http://imss.gob.mx/) revisada el 22 de marzo del 2011.

54.- Habicht, JP. Estandarización de métodos epidemiológicos cuantitativos sobre el terreno. Washington; 1974. p. 62-65.

55.- WHO, Obesity preventing and managing the global epidemic report of a WHO consultation on obesity. 1998, Geneva, 3-5 June, 1997.

56. Griffith OW. Determination of glutathione and glutathione disulfide using glutathione reducíase and 2-vinylpyridine. Anal Biochem 1980; 106: 207-12.

57. Bradford M. 1976. A Rapid Sensitive Method for the Quantitiation of Microgram Quantities of Protein utilizing the principle of Protein Dye-Binding. Analytical Biochemistry 72: 248-254.

58.- Madrigal-Fritsch H, Martínez-Salgado H. Manual de encuestas de dieta. 3ra ed. México: Instituto Nacional de Salud Pública; 1996. p. 27-32.

59.- Baril JG, Kovacs C, Trottier S, et al. Effect of a low dose of salmon oil on triglycerides and lipid parameters in HIV patients receiving a HAART regimen and having elevated serum triglyceride levels and total cholesterol/high density lipoprotein ratio. 13^th^ Conference on Retroviruses and Opportunistic Infections; February 5-8, 2006; Denver. Abstract 756.

**INSTITUTO MEXICANO DEL SEGURO SOCIAL**

**Appendixes**

**Appendix 1.** Written informed consent for participation in the protocol**:**

**EFfect of**

**omega 3 acids ON OXIDATIVE STRESS in seropositive HIV patients**

Leon, Guanajuato ____________ 20___

We invite you to participate in a research study with register number ____________ that will take place in the Clinical Research Unit of the Medical Unit of High Specialty No.1 Bajio. Your participation is voluntary, and you can withdraw at any time you choose without the medical care received in the Mexican Social Security Institute is affected.

Our purpose is that you receive the correct and sufficient information to help you decide whether to participate in the project. To do this we ask that you carefully read this informed consent sheet, we will clarify any doubts that may arise after reading the document. You are free to consult with persons it deems necessary.

This form may contain words or concepts you do not understand, please ask the researcher doctor to explain any words or information that you do not understand. You can take home an unsigned copy of this consent to discuss it with your family or with your doctor before taking its decision.

**Objective of the study.**

The aim of this study is to know the effect of using liquid fats known as omega-3 on a process known as "oxidative stress" that has been linked to disorders such as heart disease associated with your condition and medications required to control your disease.

You have been invited to participate in this study where we will make measurements of weight and height, we need you answer a survey data related to feeding and undergo blood tests that are part of the same monitoring control of your disease in addition to also determine some oxidative stress substances in your blood. It is also likely that you remain assigned to receive a food supplement containing oils known as omega 3 fatty acids or by chance a placebo that has a similar presentation but is not pharmacologically active. We believe that you are a good candidate because your cholesterol and triglyceride levels are above normal. Like you, 70 others will be invited to participate.

We reiterate that participation in this study is completely voluntary and you can withdraw at any time moment without this causes changes in your care service. Please read the information that we provide to you and ask any questions you wish before deciding whether or not to participate.

**Procedures**

If you agree to participate in the study will be asked to sign this consent form, your doctor will gather information about your disease and start assessing.

Your participation in the study will last about six months and will include 4-5 visits.

At Visits 1 and 4 or 5 dietary surveys will be conducted: 24-hour recall and food diary to know how are your food habits. Also we will take a sample of blood from your arm about 15 ml.

Anthropometric and clinical tests that will make you every one of the visits include measurement of weight, height, waist, hips, and blood pressure measurement, as well as your physician will evaluate you do every day in each of your visits considering physical examination and clinical manifestations related to your illness. By chance you may be invited to belong to the placebo group or can receive a jar with tablets containing omega 3 as a food supplement and on each visit plus the number of tablets ingested you will be counted. On average you will have an appointment every two months unless your treating physician considers that requires more frequent evaluation and schedule their visits will be recorded on your card and in accordance with the programming of your treating physician.

**Potential risks and discomforts.**

The risks associated with clinical evaluation procedures (measurement of weight, height, waist, hip, blood pressure) are noninvasive studies that do not cause pain, discomfort or risk. Discomfort during blood sampling are minimal, sometimes the procedure may cause some pain or form a bruise. By administering the food supplement that contains known oils such as omega 3 fatty acids you may experience a "fishy taste", and alterations in intestinal transit as gas or diarrhea and although it has been reported to decrease the generation of a coagulation protein of blood. Hoverer; it has not been observed increased risk of bleeding, but in any case we will be monitoring the adverse effects that may arise from your participation in the study and measures required to resolve them will be taken. Allergic reaction associated with the drug or elevated enzymes produced in the liver will also be monitored during follow-up.

**Potential benefits you will get to participate in the study.**

If you agree, alterations oxidation of blood can be detected early and then prevent cardiovascular disease as a result of late diagnosis.

Part of your participation in this study may contribute to the implementation of a test that can help you and other patients in the future and to identify alternative treatments to reduce the risk of death from heart or vascular cause.

One benefit of participation is that the results will make your knowledge and so you really know how your health is and can perform other preventive actions.

**Compensation for injuries.**

Your participation in the study will not cause you any expense. The Mexican Social Security Institute will provide laboratory studies and ultrasound at no cost to you. No payment will be made to participate in this study.

**Results or new information on diagnostic alternatives.**

During the course of this study, we will inform you of any information obtained is important for the decision to participate in the project.

**Participation or withdrawal.**

Your participation in this study is completely voluntary. If you decide not to participate in any way you will receive the medical care you usually receive in this institution. That is, it will not affect your relationship with the IMSS and will not affect your right to health care or other services. You can ask the questions you want to start the project and along the people in charge of the study.

**Privacy and confidentiality.**

The information you provide that could be used for identification (as the name, address and telephone) will be kept confidential and separate as well as your responses to the questionnaires and the results of clinical tests, to ensure your privacy.

The research team, your doctor and the people who are involved in your care know you are participating in this study. However, no one else will have access to the information you provide while participating in this study, unless you so desire. Just provide your information if necessary to protect your rights or welfare, or if required by law.

When the results of this study are published or presented at conferences, for example, we will not give information that could reveal your identity. Your identity will be protected and hidden. To protect your identity we will assign a number to use to identify your data, and will use that number instead of your name in our database.

**Personal contact for questions and clarifications about the study.**

If you have questions or want to talk to someone about this research study can communicate from 7:00 to 15:00 h, Monday to Friday with Dr. Norma Amador Licona, who is the researcher responsible for the study, the phones: 7 17 48 00 ext. 31315 and you can also check with your physician.

In case of emergency a derivative of the study, you can dial the phone: 044 477 1265759 at any time of day, any day of the week.

**Personal contact for questions about your rights in a research study**.

If you have questions about their rights to participate in a research study, you may contact the Commission responsible for Research Ethics IMSS to Telephone number: 56276900-21216, 9 to l6:00 h; or if you prefer to email: conise@cis.gob.mx. The Ethics Commission is located in el Edificio del Bloque B, Unidad de Congresos piso 4, Centro Medico Nacional Siglo XXI, Av. Cuauhtémoc 330 Colonia Doctores, C.P. 06725, Mexico D.F.

Do not sign this information sheet and informed consent form unless you have had the opportunity to ask questions and received satisfactory answers to all your questions.

**Informed consent statement**

I have been clearly explained about this study, I have also read (or someone has read to me) the content of this consent form. I have been given the opportunity to ask questions and all my questions have been answered to my satisfaction. It has given me a copy of this form.

By signing this form I agree to participate in this research.

_________________________________________________

Name of the participant

_____________________________ _____________________________

Sign of the participant Date

**Signature responsible for obtaining informed consent**

I have explained the research study to the participant and have answered all his/her questions. I think he/she understood the information described in this document and freely consent to participate in this research study.

__________________________________________________

Name charge of obtaining informed consent

________________________________ _____________________________

Signature charge of obtaining informed consent Date

**Signature of witness**

My signature certifies that the participant signed this form before me informed consent voluntarily.

_____________________________________ __________________________

Name of witness 1 Relationship to participant

**Adress____________________________ Tel.:____________________**

_______________________________________ __________________________

Signature of witness Date

________________________________________ ___________________________

Nane of witness 2 Relationship to participant

______________________________________ ___________________________

Signature of witness Date

**Adress____________________________ Tel.:____________________**
